# Genomic Regions Analysis of Seedling Root Traits and Their Regulation in Responses to Phosphorus Deficiency Tolerance in CSSL Population of Elite Super Hybrid Rice

**DOI:** 10.3390/ijms19051460

**Published:** 2018-05-14

**Authors:** Galal Bakr Anis, Yingxin Zhang, Huimin Wang, Zihe Li, Weixun Wu, Lianping Sun, Aamir Riaz, Liyong Cao, Shihua Cheng

**Affiliations:** 1 State Key Laboratory of Rice Biology, China National Rice Research Institute, Hangzhou 310006, Zhejiang, China; galalanis5@gmail.com (G.B.A.); zyxrice@163.com (Y.Z.); lizihe0820@163.com (Z.L.); wuweixun@caas.cn (W.W.); slphongjun8868@126.com (L.S.); aamirriaz33@gmail.com (A.R.); 2 Rice Research and Training Center, Field Crops Research Institute, Agriculture Research Center, Kafr Elsheikh 33717, Egypt; 3 Jiangxi Academy of Agricultural Sciences, Nanchang 210014, China; wxn6700@163.com

**Keywords:** rice, phosphorus deficiency, genomic regions, CSSLs, root traits

## Abstract

Phosphorus (P) is the essential macro-element supporting rice productivity. Quantitative trait loci (QTL) underlying related traits at the seedling stage under two different phosphorus levels was investigated in rice using a population of 76 Chromosomal Sequence Substitution Lines (CSSLs) derived from a cross between the maintainer variety XieqingzaoB (P stress tolerant) and the restorer variety Zhonghui9308 (P stress sensitive); the parents of super hybrid rice Xieyou9308. A genetic linkage map with 120 DNA marker loci was constructed. At logarithmic odd (LOD) value of 2.0, a total of seven QTLs were detected for studied traits under two P levels and their relative ratio. The LOD values ranged from 2.00 to 3.32 and explaining 10.82% to 18.46% of phenotypic variation. Three QTLs were detected under low phosphorus (P^−^), one under normal (P^+^) and three under their relative ratio (P^−^/P^+^) on the rice chromosomes 3, 5, 6, 8 and 10. No significant QTLs were found for shoot dry weight (SDW) and total dry weight (TDW). The pleiotropic QTLs influencing root number (*qRN5*) and root dry weight (*qRDW5*) as novel QTLs under P^−^ level were detected near marker RM3638 on chromosome 5, which considered to directly contributing to phosphorus deficiency tolerance in rice. These QTLs need further analysis, including the fine mapping and cloning, which may use in molecular marker assisted breeding.

## 1. Introduction

Rice (*Oryza sativa* L.) is grown in many parts of the world and considered as a primary staple food source for more than half of the world’s population. Therefore, rice plays an important role to boost the food security and poverty alleviation in rice-eating countries, although it is cultivated in areas with soil phosphorus deficiency [1]. Nitrogen (N), phosphorus (P) and potassium (K) are the three most common and primary nutrients required for plant growth and development, which are applied to rice fields in the form of chemical fertilizers [2]. The limitation of soil fertility and high costs of fertilizers are the biggest challenges faced by the farmers in most of rice cultivated areas [3]. Phosphorus (P) is fundamental to crop development because it’s playing main roles as a component of many organic molecules, phospholipids and proteins [4,5]. Generally, phosphorus as one of the three nutrients added to soil because it is readily leached by soil particles [6]. Approximately 80% of phosphorus content in the soil can be fixed in non-available forms to plants [7]. Beside yield as the most important trait, studying of root growth parameters such as root length, root number, root dry weight, shoot dry weight and total dry weight under various stress conditions are the important traits in rice breeding programs. Most of the stresses in rice lead to changes in root system architecture and phosphorus (P) deficiency is one of them [8,9,10]. Interestingly, P deficiency is one of the main limiting factors in rice production in many rice cultivated countries. Screening and developing rice genotypes that have the properties of phosphorus (P) efficiency provide an alternative and promising solution to resolve this problem, through the utilization of those genotypes as parents in the rice breeding programs [11,12].

Genomic technologies, molecular markers as well as statistical methods had revolutionized the genetic analysis of plant breeding and provide valuable tools for identifying genomic regions influencing abiotic stresses such as phosphorus deficiency tolerant. These technologies have led to a growing understanding of the underlying processes behind plant responses to the P deficiency of molecules through the whole plant level [13]. Marker technologies and maps allow the quantitative trait loci (QTL), site genomic regions with positive effects on yield stability, or phosphorus deficiency tolerant under adverse environmental conditions [13]. Mapping Quantitative trait loci (QTL) are a powerful method of analysis to assign the positions of genes contributing to seedling root traits related to P deficiency tolerance in rice. Previous studies associated with P deficiency in rice seedling stage identified different QTLs and reported that a P deficiency is controlled by complex quantitative traits [14,15,16,17,18,19]. The genome mapping can be used to identify the QTLs associated with phosphorus deficiency traits, controlled by multiple genes and show the genetic characteristics of the quantitative properties [11,18,20,21,22,23]. A total of those studies investigated root traits performance under P deficient conditions [14,18,24,25]. Several QTLs for low phosphorus tolerance in rice has been detected on chromosomes 1, 2, 3, 4, 5, 6, 9, 10 and 12 [26]. *PSTOL1* as a major candidate gene for phosphorus (P) deficiency was located on chromosome 12 of rice and cloned from P deficiency tolerant rice variety Kasalath under name *Pup1* [27,28]. The information of loci associated with traits under phosphorus deficiency is important to develop the new rice varieties with the stress tolerance.

In our study, a rice population consisting of 75 CSSLs, derived from a cross between XieqingzaoB (P deficiency tolerance) and Zhonghui9308 (P deficiency susceptible), were used. This study investigated the effects of P deficiency treatment and detected quantitative trait loci (QTLs) for P deficiency traits at the seedling stages of rice. We examined the additive QTLs for P deficiency in root and shoot traits under two levels of phosphorus (P^+^ and P^−^) using IciMapping (Wang 2009) approaches. These QTLs can be used in improving P deficiency tolerance of rice by MAS and the results will be helpful for understanding the molecular mechanisms under phosphorus deficiency conditions.

## 2. Results

### 2.1. Phenotypic and Frequency Distribution of 75 CSSLs and Their Parents

**Figure 1:**
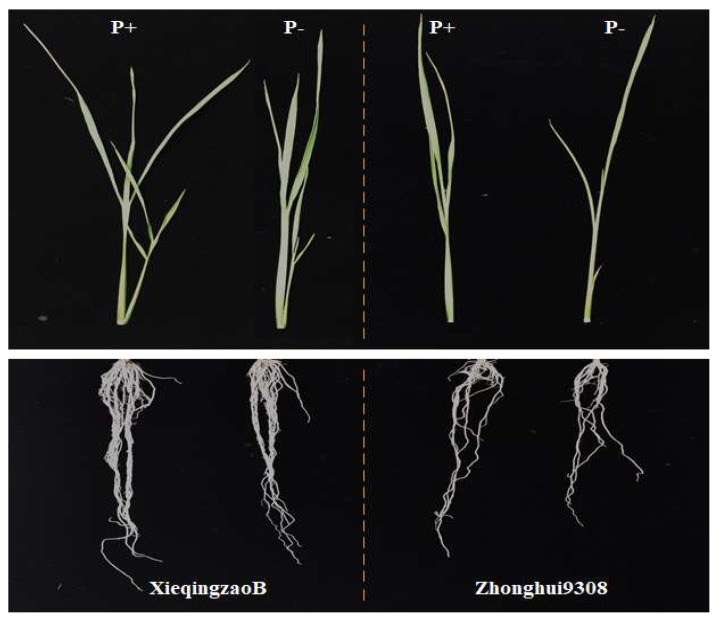
Phenotype of the rice parental lines XieqingzaoB and Zhonghui9308 after four weeks under hydroponic conditions, P^+^ normal conditions and P^−^ deficient conditions.

**Figure 2:**
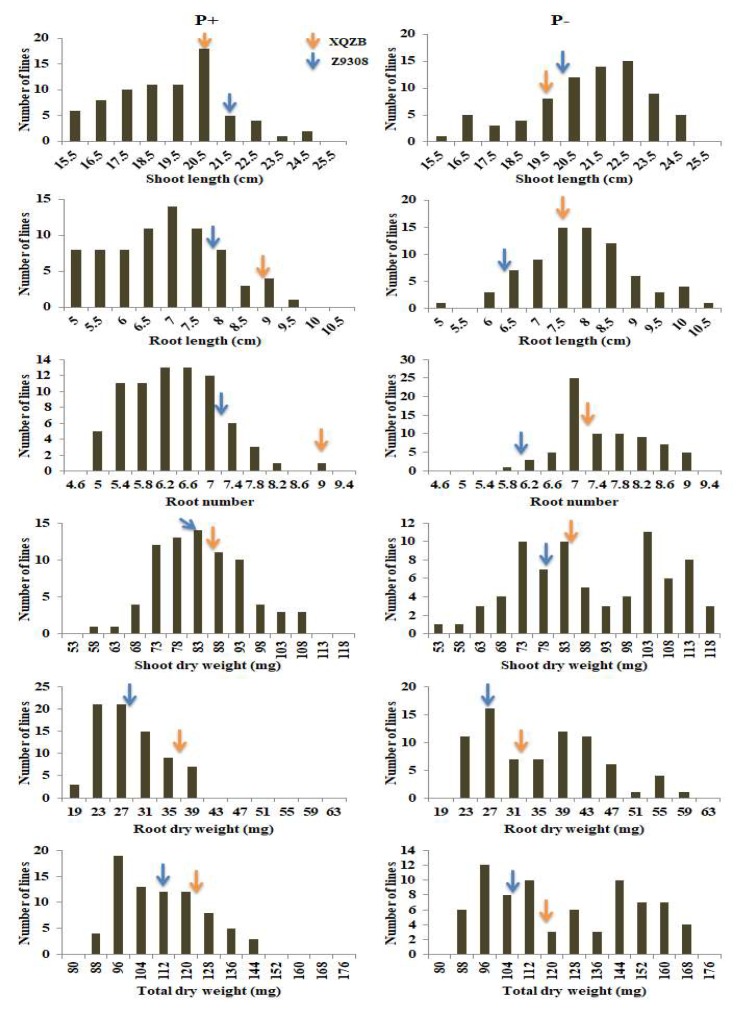
Distribution of mean values of 75 CSSLs and their parents for six seedling root traits under two phosphorus levels, normal (P^+^) and low (P^−^). Red and blue arrows indicate the mean values for XieqingzaoB (XQZB) and Zhonghui9308 (Z9308), respectively.

The phenotypic variation and distributions of six seedling traits of the two parents and the CSSL population under both deficient and normal P conditions are shown in Table 1. The mean performance values of each parameter are given across treatments and separately for the normal and low phosphorus levels. Among the six studied traits at the seedling stage, the parent XieqingzaoB recorded higher mean performance values than those in Zhonghui9308 for all traits except shoot length trait under both P levels, indicating that XieqingzaoB was integrally more tolerant than Zhonghui9308 to phosphorus deficiency at rice seedling stage (Table 1 and Figure 1). Wide variations for all six seedling traits were observed under both the normal and low P treatments, and the transgressive segregations were positive under both P levels except root number (RN) under normal P level. The kurtosis and skewness for the distributions of the studied six traits in the chromosomal segment substitution lines (CSSLs) population were less than unit except shoot dry weight (SDW) and total dry weight (TDW), suggesting that these traits near normal distributions and the data is suitable for quantitative trait loci analysis. Moreover, these traits illustrated frequency distribution, genetic characteristics of quantitative traits, existed transgressive segregation and polygenic inheritance (Figure 2). For analysis of variance, two-factor ANOVA (genotype and treatment) indicated that genotype, treatment, and genotype × treatment interaction were significantly different in CSSL population (Table 2). The effects of the genotypes and genotype-by-environment interaction were almost equally important for all traits except genotypes for the root number trait.

### 2.2. Correlation Coefficient Analysis among Seedling Root Traits

In our investigation, we get insight in the phenotypic correlation coefficient analysis between the six seedling traits separately; one with line means from normal P and one with line means from low P and the results are presented in Table 3. According to the results, significant positive correlation coefficients were observed between all studied traits under normal P. Similar correlations were also found under low P. These correlations ranged from 0.322 to 0.967 under normal P, and from 0.242 to 0.971 under low P (Table 3). Under both the normal and low P treatments, TDW recorded the strongest correlations with SDW and RDW with values of 0.967 and 0.849 under normal P and 0.971 and 0.912 under low P, respectively. Simultaneously, a significant positive correlation was also observed in the SL trait between the normal P and low P unlike RN and RDW where had recorded significant negative correlations.

### 2.3. QTL Analysis for Seedling Root Traits under P^+^ and P^−^ Levels and Their Relative Ratio

**Figure 3:**
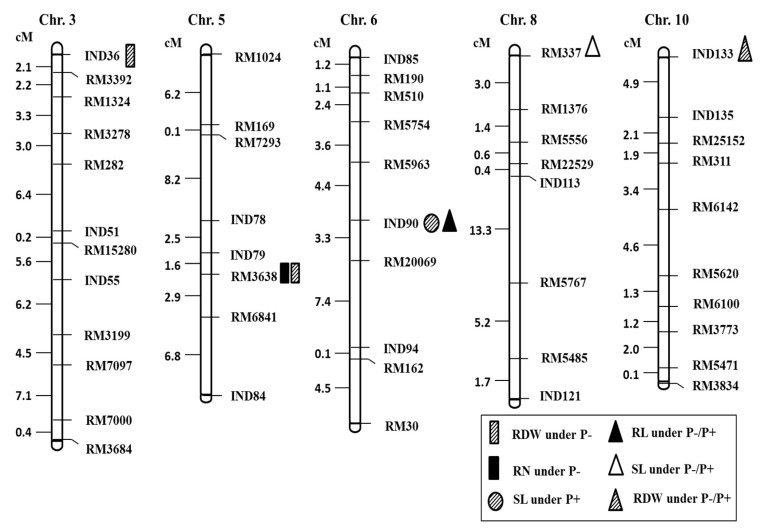
Chromosomal location of putative QTLs for root dry weight (RDW), root number (RN), shoot length (SL) and root length (RL) under both the normal (P^+^) and low (P^−^) phosphorus and their ratio (P^−^/P^+^) conditions.

A total of seven QTLs were detected with significant LOD values for each trait under the two P levels and their ratio (Figure 3). Among six seedling traits, no significant QTLs were found for shoot dry weight (SDW) and total dry weight (TDW). For seven QTLs were detected under P^+^, P^−^ and P^−^/P^+^, the LOD values ranged from 2.00 to 3.32 (Table 4). QTL explained the phenotypic variance ranged from 10.82% to 18.46%. Three QTLs were detected under P^−^, one under P^+^ and three under P^−^/P^+^ ratio. The QTLs were distributed on the rice chromosomes 3, 5, 6, 8 and 10; most of them are one QTL on chromosome 3, two QTLs on chromosome 5, two QTL on chromosome 6, one QTL on chromosome 8 and one QTL on chromosome 10 were detected (Figure 3). Two QTLs of shoot length (SL) were detected on chromosomes 6 and 8. One QTL, *qSL6* was detected near marker InD90 under normal phosphorus (P^+^) level, and the other QTL, *qSL8* was detected near the marker RM337 under P^−^/P^+^ ratio. The explained phenotypic variance and LOD scores of two QTL were 16.94% and 3.02 and 18.46% and 3.32, respectively. There was only one QTL (*qRL6*) for root length (RL) that was mapped under P^−^/P^+^ ratio by IciMapping. The QTL *qRL6* was located on chromosome 6 near the marker InD90, with a LOD score of 2.31 that explained 16.07% of phenotypic variance which was the same region harboring a QTL for shoot length under normal phosphorus P^+^ level. The positive allele for *qRL6* was contributed by XieqingzaoB and this contributed to increase the root length trait. For root number trait, only one QTL (*qRN*5) under low phosphorus (P^−^) was identified on chromosome 5 in the marker RM3638 with LOD value of 2.21 and explaining phenotypic variation of 12.71%. The positive allele for *qRN*5 was contributed by XieqingzaoB. Concerning the QTLs for root dry weight (RDW), three QTLs for this trait were revealed under two different phosphorus (P) levels. Under low phosphorus (P^−^) level, two QTLs were detected. One QTL (*qRDW3*) controlling root dry weight was located on chromosome 3 in the marker interval InD36, with a LOD score of 2.00 that explained 10.82% of phenotypic variance. Under the same P level, another QTL (*qRDW5*) was detected on chromosome 5 which was the same region harboring a QTL for the root number under P^−^ with explaining about 12.66% phenotypic variation and LOD score of 2.37. Simultaneously, one QTL (*qRDW10*) was located on chromosome 10 near marker InD133 under P^−^/P^+^ ratio with a LOD value of 2.18 that explained 12.52% of phenotypic variance. The additive effects were positive in *qRDW5* and *qRDW10*, implying the alleles were derived from the XieqingzaoB parent line increased the root dry weight. On the other hand, the additive effect was negative in *qRDW3* indicating that, the allele derived from Zhonghui9308 of this QTL was responsible for decreasing root dry weight in populations.

### 2.4. Seedling Root Traits in CSSLs under P deficiency

**Figure 4:**
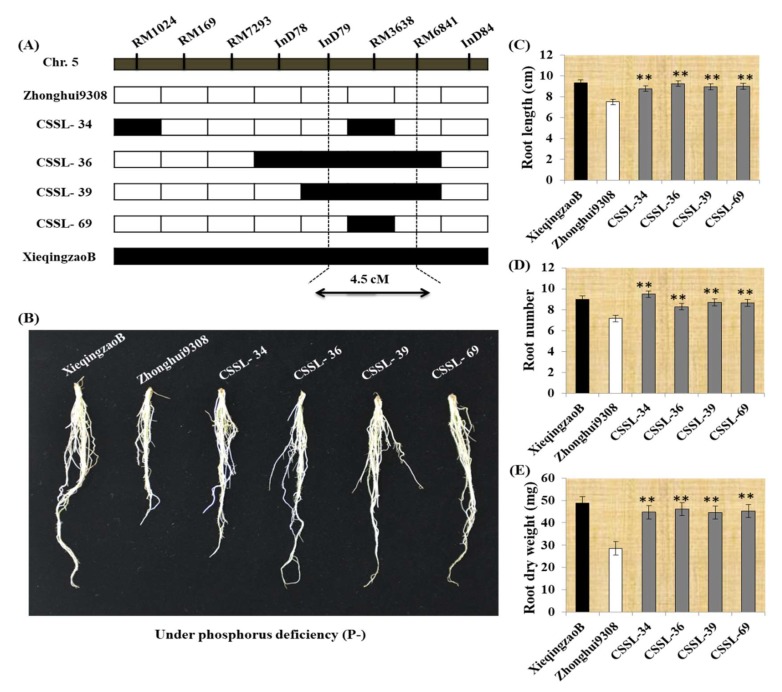
Graphical genotypic and phenotypic of CSSL-34, CSSL-36, CSSL-39 and CSSL-69 and their parents. (**A**) Putative QTL region for root number and root dry weight under P deficiency on rice chromosome 5. (**B**) Phenotypic variation between CSSLs and parents. (**C**–**E**) Measurements of root length, root number and root dry weight of XieqingzaoB, Zhonghui9308 and CSSL lines. The asterisks represent statistical significance between CSSLs and Z9308, determined by a student’s *t*-test (** *P* < 0.01).

Using 75 CSSL populations, two co-located additive QTLs associated with root number (*qRN5*) and root dry weight (*qRDW5)* when plants were exposed to P^-^ deficiency. Four lines, CSSL-34, CSSL-36, CSSL-39 and CSSL-69 are harboring these QTLs and each carry an introgression in this region and carry a common XieqingzaoB derived segment on the long arm of chromosome 5 (Figure 4A). To inform the root behavior of these lines under P^-^ deficiency compared to the parental lines, an additional experiment for 40 days was done under P^-^ deficiency. Therefore, the data presented in (Figure 4C–E) were taken from 40 day old seedlings under phosphorus deficient conditions. These four lines showed significantly higher results in root traits compared with Zhonghui9308 as a genetic background parent (Figure 4B). The candidate region was delimited between InD79 and RM6841 as neighboring markers of these QTLs on the long arm of chromosome 5 (Figure 4A), and corresponded to the QTL detected in the CSSL population. The physical length of the candidate region was approximately 4.5 cM. Concerning the three root traits, root length, root number and root dry weight, the four CSSLs gave significantly higher compared with Zhonghui9308 under P^-^ deficiency and was similar to that of the corresponding genetic background, Zhonghui9308 (Figure 4C–E).

## 3. Discussion

Quantitative trait loci (QTL) mapping is a powerful tool for genetic analysis of complex quantitative characters and a good way to detect the genes contributing to root seedling traits related to phosphorus deficiency tolerance in rice [18,22]. Detection of QTLs for seedling traits, like root length, root number, root dry weight and shoot dry weight can give novel insights into crop breeding to tolerate biotic and abiotic stresses [29]. Numerous investigations have been carried out to identify the QTLs for root traits associated with different phosphorus (P) levels using various mapping populations [11,14,15,16,17,18,21,22,23,30,31,32]. By using 271 introgression lines of Yuefa/IRAT109 at the seedling stage, Li et al. [18] detected 11 additive QTLs and 15 pairs of epistatic QTLs under P deficiency, while seven additive QTLs and nine pairs of epistatic QTLs were identified under P-sufficiency condition. In our investigation, to understand the genetic basis and identify the QTLs associated with seedling growth traits, we evaluated 75 CSSLs previously developed from XieqingzaoB/Zhonghui9308 under two phosphorus (P^+^ and P^−^) levels. The lines utilized in present study were homozygous with segment from the donor parent XQZB. The parent XieqingzaoB recorded higher mean performance values than those in Zhonghui9308 for all traits except shoot length trait under both P levels, indicating that XieqingzaoB was integrally more tolerant than Zhonghui9308 to phosphorus deficiency at rice seedling stage consistent with previous findings [23]. Based on the traits data, we performed QTL analysis for six seedling traits using 87 SSR and 33 InDel markers. Each line was genetically fixed and had the genetic background of XieqingzaoB. Furthermore, the transgressive segregation was observed for most of the studied traits (Figure 2) indicating that the donor parent (XieqingzaoB) carried favorable genes of seedling traits under phosphorus deficiency.

Rice genotypes differed in their response to phosphorus deficiency, and the observed differences were attributed to genetic variation. Consequently, shoot length is an important indicator of the plant response to P^−^ related stress. Two QTLs of shoot length (SL) were detected on chromosomes 6 and 8, whereby *qSL6* was located near the InD90 marker under normal phosphorus (P^+^) level, and *qSL8* was detected near the marker RM337 under P^−^/P^+^ ratio. The explained phenotypic variation of the two QTLs was 16.94% and 18.46%, respectively. By using DH population of CT9993 × IR62266 cross, Kanbar et al. [33] identified the QTL associated with root and related traits and found two QTLs (*qpht8-1*, *qpht8-2*) for plant height in chromosome 8 with the respective LOD values of 3.20 and 2.45 and explained phenotypic variance of 12.6% and 8.5%. Wang et al. [34] performed the QTL analyses associated with rice root traits in hydroponic conditions using 215 recombinant inbred lines derived from the same parents (ZQXB and Z9308) as those used in the present study, and identified three QTLs (*qPH7*, *qPH8* and *qPH10*) affecting plant height. Lin et al. [19] evaluated a set of recombinant inbred lines (RILs) for five seedling growth traits that emerge in P^−^ deficient conditions. The authors detected twenty QTLs for seedling growth response to P deficiency on chromosomes 1, 4, 5, 7, 8, 9, 11 and 12, none of which correspond to the QTLs detected in this investigation. This discrepancy could be due to the differences in the parent cultivar combinations or variations in culture conditions. In a similar study of recombinant inbred line (RIL) population derived from the super hybrid rice XQZB/Z9308, in which effects of two nitrogen levels were examined, Feng et al. [35] detected QTLs for plant height under both nitrogen levels on chromosomes 6, which explained 15.85% and 13.14% of the observed phenotypic variance. Root length is the most important trait for P deficiency because roots provide an interface between the plant system and various soil stresses. In our investigation, one QTL (*qRL6*) for root length was detected under P^−^/P^+^ ratio on chromosome 6 near the marker InD90, with an LOD score of 2.31, which explained 16.07% of the observed phenotypic variance. The QTL *qSL6* for shoot length under normal phosphorus P^+^ level was located in the same region. The positive allele for *qRL6* was contributed by XieqingzaoB, which accentuated the root length trait. Niu et al. [36] reported that, phosphorus deficiency in rice leads to increased root length. Similarly, *qREP-6*, a root elongation QTL under P- deficient conditions was mapped on chromosome 6 [18,21,37]. Moreover, *qRL6.1* as a major QTL for root length under growth conditions characterized by different nitrogen concentrations at the seedling stage was mapped by Obara et al. [38]. However, it is noteworthy that the QTLs identified by different authors were not located in the region where Zhang et al. [39] detected three QTLs for root length (*qRRL7*, *qRRL11* and *qRRL12*) under P-deficient growth conditions, for which an LOD value of 2.91, 2.16 and 2.30 was reported, along with 13.3%, 10.7% and 10.3% total phenotypic variation explained, respectively. In addition, Zhang et al. [23] mapped two QTLs, *qTPDE7.1* and *qTPDE7.1* for root length under P-deficient conditions at the tillering stage. Wang et al. [34] performed QTL (*qRL7*) on chromosome 7 between markers RM3859 and RM214, with 18.14–18.36% phenotypic variance explained. Previous findings suggest that the number of roots is the most important growth factor for ensuring that the plant obtains the required nutrients from soil, such as P, especially when subjected to environmental stresses [40]. When examining the number of roots as a specific trait, only one QTL (*qRN*5) was detected under low phosphorus (P^−^) conditions. It was located on chromosome 5 near the marker RM3638 with an LOD value of 2.21 and 12.71% phenotypic variation explained. It is noteworthy that, one QTL for root dry weight (*qRDW5*) under the same P level was also identified in this region. The correlations between these two QTLs (*qRN5* and *qRDW5*) were statistically significant irrespective of the P level, indicating that these traits promote phosphorus deficiency tolerance in rice cultivars. *qRN5* and *qRDW5* are novel QTLs for P deficiency that have not been reported in extant studies, in which no QTLs for these traits have been found in this region. Li et al. [18] detected two QTLs (*qRN4* and *qRN8*) for root number under P-deficient growth conditions. For the root dry weight (RDW), two QTLs were detected in plants grown in low phosphorus (P^−^) conditions. One QTL (*qRDW3*) was detected on chromosome 3 near the marker InD36, accounting for 10.82% of the observed phenotypic variance, while the other QTL (*qRDW5*) was identified on chromosome 5, near the marker RM3638. In this region, a QTL for the number of roots as a function of the P level was identified, which explained about 12.66% of the total phenotypic variation. On the other hand, one QTL (*qRDW10*) was identified on chromosome 10 near the marker InD133 under P^−^/P^+^ ratio, accounting for 12.52% of the total phenotypic variance. Some of these QTLs related to the RDW trait were new, while other were matched to the QTLs of RDW and related traits reported in the previous studies.

The application of QTL mapping for low phosphorus tolerance may greatly facilitate improvement of P deficiency tolerance in rice. We considered the QTLs identified on chromosome 5 to be the most effective because these QTLs were detected under P deficiency level with positive additive effect. Between the secondary physiological indices of phosphorus deficiency, the root number and root dry weight are more important than others. The QTLs, *qRDW5* and *qRN5* as novel QTLs located in the same region, indicate the strong relationship between these traits especially under P deficiency and their ability to increase root system may contribute to P deficiency tolerance. Out of 75 CSSLs, four lines CSSL-34, CSSL-36, CSSL-39 and CSSL-69 each carry an introgression in this region on chromosome 5, and all had significantly root length, root number and root dry weight than Zhonghui9308 when grown under P deficiency (Figure 4C–E). These lines would be good to select for further study on the basis of a genetic map of the 75 chromosome segment substitution lines. In conclusion, the detection of new QTLs associated with P deficiency tolerance will give confirmed information for a genetic basis and functional analysis of rice P^−^ tolerance, because they were identified in the CSSL population derived from two different parents. The molecular markers that are nearest to the QTLs will be useful for Marker Assisted Selection (MAS) to develop new rice varieties/lines with a high level of P deficiency tolerance in rice breeding program. In addition, the P deficiency tolerance lines may be used as new materials for rice breeding program.

## 4. Materials and Methods

### 4.1. Plant Materials

**Figure 5:**
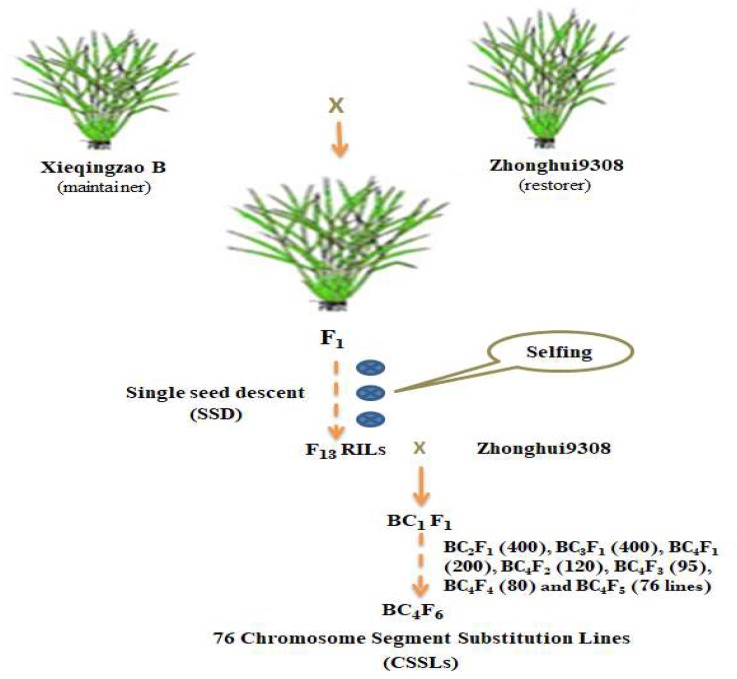
Crossing scheme for developing the 76 chromosome segment substitution lines (CSSLs).

A hybrid population, Chromosomal Segment Substitution Lines (CSSLs) derived from a cross between the P stress tolerant variety XieqingzaoB (maintainer) and the P stress sensitive variety Zhonghui9308 (restorer) as parental lines for super hybrid rice (Xieyou9308) in China. This CSSLs population consisted of 76 lines carrying XieqingzaoB chromosomal segments on a genetic background of Zhonghui9308. The CSSL populations were developed as shown in Figure 5. The substituted chromosome segments covered most of the 12 chromosomes by 76 CSSLs. As one line was removed from this population for the lack of seeds, 75 lines plus their parents were used in this study.

### 4.2. Hydroponic Experiment

Seeds of 75 Chromosomal Segment Substitution Lines (CSSLs) and two parents were soaked for three days at 28 °C in water. Fifteen germinated seeds were sown on foam plates floated in pools and grown for one week before being moved to the containers. The experiment was conducted in the greenhouse condition at China National Rice Research Institute (CNRRI) at Hangzhou, China, from March 2017 to January 2018. The experiment design consisted of randomized complete blocks with three replications, five plants for each replicate. During the experiment, the plants were raised under the following conditions: temperature 30 ± 3/21 ± 2 °C day/night, relative humidity 70% and natural daylight. The nutrient solution was used according to Yoshida [41] with some modifications. The culture solution was prepared in distilled water. The nutrient solution was renewed weekly, and the normal culture solution was mixture of (mg/L); 48.2 (NH_4_)_2_SO_4_, 59.9 Ca(NO_3_)_2_, 24.8 KH_2_PO_4_, 18.5 KNO_3_, 15.9 K_2_SO_4_, and 65.9 MgSO_4_ and 1 mL/L Fe-EDTA, H_3_BO_3_, CuSO_4_·5H_2_O, ZnSO_4_·7H_2_O, MnCl_2_·4H_2_O, and H_2_MoO_4_·H_2_O. For the low phosphorus treatment, the KH_2_PO_4_ concentration decreased to 0.32 (mg/L).

### 4.3. Phenotypic Investigation

The growth parameters for 75 CSSLs and the two parents were measured after four weeks from the beginning of the low P treatment. Plants were sampled for trait measurements and genotypic analysis. After they had been sampled, the total roots were separated from the shoot base and shoot length (SL), root length (RL) and root number (RN) were investigated manually, while shoots and roots were over dried at 70 °C until a constant weight to evaluate shoot dry weight (SDW), root dry weight (RDW) and total dry weight (TDW). The phosphorus treatments were marked P^+^, P^−^ and P^−^/P^+^ for control level, low level and their relative ratio, respectively. All the traits investigated in this study were summarized in Table 5.

### 4.4. QTL Mapping and Data Analysis

A total of six phenotypic seedling traits (Table 1) represented by the means values for each genotype, were used for phenotypic and QTL analysis. Statistical and phenotypic correlation coefficient analyses were calculated with SAS software version 9.2. The relative values under low phosphorus (P^−^) and corresponding value under normal (P^+^) were calculated for the QTL analysis. In our investigation, we carried out 87 SSR and 33 InDel markers, which were distributed along the rice genome according to previously reported linkage maps, and known polymorphisms between the parents, were used to determine the genotype of the CSSL populations. QTL analysis was performed with the IciMapping 4.0 (Wang, 2009) approaches. It was considered as a major effect QTL when its LOD was larger than 2.0. QTLs were named according to standard conventions of nomenclature (McCouch 2008).

## 5. Conclusions

Phosphorus (P) is an essential plant nutrient for rice growth and phosphorus deficient stress for rice occurs frequently and its depletion causes limit rice growth, agronomical loss and reduce yields. In this study and by using 75 CSSLs derived from XieqingzaoB/Zhonghui9308, a total of seven QTLs were detected for seedling root traits under normal phosphorus (P^+^), low phosphorus (P^−^) and their relative ratio (P^−^/P^+^). Three QTLs were detected under P^−^, one under P^+^ and three under P^−^/P^+^ ratio on the rice chromosomes 3, 5, 6, 8 and 10. Two QTLs influencing root number (*qRN5*) and root dry weight (*qRDW5*) as novel QTLs under P^-^ deficiency conditions and not previously reported. These QTLs are valuable for future map-based cloning, which may use in molecular marker assisted breeding for developing new rice varieties/lines tolerant to phosphorus deficiency.

## Figures and Tables

**Table 1:** Phenotypic variation for seedling root traits under both P levels in CSSLs population and their parents.

Treat.	Trait	Parents		*p*-Value	Chromosomal Segment Substitution Lines (CSSLs)					
		XieqingzaoB	Zhonghui9308		Min.	Max.	Mean	SD%	Kurtosis	Skewness
Normal phosphorus (P^+^)	SL	20.68	21.67	0.172	16.52	27.82	21.06	2.02	0.79	0.12
	RL	8.93	7.70	0.071	4.43	9.23	6.56	1.14	−0.58	0.20
	RN	9.00	7.17	0.022	5.00	8.67	6.18	0.80	−0.19	0.47
	SDW	86.43	83.83	0.007	55.38	104.8	80.91	10.64	−0.25	0.27
	RDW	37.22	28.00	3.710	18.33	39.0	26.59	5.13	−0.46	0.64
	TDW	123.65	111.83	0.000	82.01	142.1	107.51	14.65	−0.47	0.48
Low phosphorus (P^-^)	SL	19.46	20.29	0.166	15.03	24.33	20.48	2.19	−0.25	−0.52
	RL	7.54	6.34	0.071	4.37	10.33	7.62	1.08	0.39	0.07
	RN	7.35	6.00	0.051	5.67	9.50	7.36	0.79	−0.37	0.33
	SDW	84.33	79.00	0.001	50.00	115.67	87.39	16.81	−1.10	−0.08
	RDW	32.67	26.67	0.029	19.38	59.00	33.79	9.84	−0.59	0.46
	TDW	117.00	105.67	2.36	82.67	167.7	121.19	25.29	−1.35	0.14

Abbreviations: SL: Shoot length (cm); RL: Root length (cm); RN: Root number; SDW: Shoot dry weight (mg); RDW: Root dry weight (mg) and TDW: Total dry weight (mg).

**Table 2:** Analysis of variance of six seedling root traits in the CSSL population of XieqingzaoB/Zhonghui9308.

S.O.V	*df*	*MS*	*F*-Value	*p*-Value
**Shoot length (cm)**				
Genotype (G)	74	14.92	17.01	0.01
Treatment (T)	1	25.61	29.31	0.34
G × T	74	2.93	33.00	0.73
Error	150	4.09		
**Root length (cm)**				
Genotype (G)	74	2.26	13.06	0.02
Treatment (T)	1	84.44	49.00	0.20
G × T	74	2.72	16.60	0.48
Error	150	1.18		
**Root number**				
Genotype (G)	74	0.70	19.35	0.11
Treatment (T)	1	102.80	28.25	0.00
G × T	74	1.85	50.87	0.08
Error	150	0.65		
**Shoot dry weight (mg)**				
Genotype (G)	74	373.95	40.15	0.76
Treatment (T)	1	3152.26	33.84	0.00
G × T	74	417.90	44.87	0.27
Error	150	113.21		
**Root dry weight (mg)**				
Genotype (G)	74	76.92	498.22	0.00
Treatment (T)	1	3889.58	100.23	0.00
G × T	74	169.65	437.00	0.00
Error	150	96.90		
**Total dry weight (mg)**				
Genotype (G)	74	691.25	57.09	0.44
Treatment (T)	1	14045.53	113.60	0.90
G × T	74	1017.78	84.06	0.63
Error	150	214.63		

**Table 3:** Correlation coefficient analysis among the studied traits under both the normal and low P treatments in the CSSLs.

Low Phosphorus (P^−^)									
**Normal Phosphorus (P^+^)**	**Traits**	**SL**	**RL**	**RN**	**SDW**	**RDW**	**TDW**	**Traits**	**Low Phosphorus (P^-^)**
	**SL**	0.673 **	0.519 **	0.394 **	0.542 **	0.242 *	0.454 **	**SL**	
	**RL**	0.322 **	−0.091	0.440 **	0.621 **	0.592 **	0.643 **	**RL**	
	**RN**	0.398 **	0.441 **	−0.449 **	0.622 **	0.592 **	0.644 **	**RN**	
	**SDW**	0.605 **	0.610 **	0.577 **	−0.061	0.786 **	0.971 **	**SDW**	
	**RDW**	0.362 **	0.646 **	0.617 **	0.686 **	−0.458 **	0.912 **	**RDW**	
	**TDW**	0.566 **	0.670 **	0.635 **	0.967 **	0.849 **	−0.220	**TDW**	
	**Traits**	**SL**	**RL**	**RN**	**SDW**	**RDW**	**TDW**	**Traits**	
**Normal Phosphorus (P^+^)**									

The data along the diagonal and shaded in black correspond to correlations between the traits under two P levels. The data above the diagonal correspond to correlations among traits under low P, while the below ones under normal P. SL, shoot length, RL, root length, RN, root number, SDW, shoot dry weight, RDW, root dry weight, TDW, total dry weight. The asterisks * and ** denote significance at the 0.05 and 0.01 probability levels, respectively.

**Table 4:** Putative QTLs controlling the seedling root traits detected in the Chromosomal Sequence Substitution Lines (CSSLs) population.

Trait	Chr.	Marker	QTL	Treatment	LOD Value	Additive Effect	PVE%	M (QQ)	M (qq)
SL	6	InD90	*qSL6*	P^+^	3.02	2.57	16.94	26.10	20.92
SL	8	RM337	*qSL8*	P^−^/P^+^	3.32	−0.11	18.46	0.76	0.97
RL	6	InD90	*qRL6*	P^−^/P^+^	2.31	0.68	16.07	2.74	1.04
RN	5	RM3638	*qRN5*	P^−^	2.21	0.62	12.71	8.54	7.29
RDW	3	InD36	*qRDW3*	P^−^	2.00	−6.13	10.82	22.51	34.77
RDW	5	RM3638	*qRDW5*	P^−^	2.37	8.01	12.66	48.95	32.94
RDW	10	InD133	*qRDW10*	P^−^/P^+^	2.18	0.81	12.52	2.92	1.30

**Table 5:** Summary of the investigated six seedling traits in this study.

Trait	Abbreviations	Unit	Trait Measurements
Shoot length	SL	cm	Measured with a ruler
Root length	RL	cm	Measured with a ruler
Root number	RN	number	Counted manually
Shoot dry weight	SDW	mg	Dried and weighted using a balance (1/1000 g)
Root dry weight	RDW	mg	Dried and weighted using a balance (1/1000 g)
Total dry weight	TDW	mg	SDW + RDW
